# Smart Driving Hardware Augmentation by Flexible Piezoresistive Sensor Matrices with Grafted‐on Anticreep Composites

**DOI:** 10.1002/advs.202408313

**Published:** 2024-11-25

**Authors:** Kaifeng Chen, Hua Yang, Ang Wang, Linsen Tang, Xin Zha, Ndeutala Selma Iita, Hong Zhang, Zhuoxuan Li, Xinyu Wang, Wei Yang, Shaoxing Qu, Zongrong Wang

**Affiliations:** ^1^ Huanjiang Laboratory School of Aeronautics and Astronautics Zhejiang University Hangzhou 310027 China; ^2^ State Key Laboratory of Silicon and Advanced Semiconductor Materials School of Materials Science and Engineering Zhejiang University Hangzhou 310027 China; ^3^ Shanghai Academy of AI for Science Shanghai 200232 China; ^4^ Institute of Thermal Science and Technology Shandong University Jinan 250061 China; ^5^ Center for X‐Mechanics Key Laboratory of Soft Machines and Smart Devices of Zhejiang Province School of Aeronautics and Astronautics Zhejiang University Hangzhou 310027 China

**Keywords:** grafted‐on composites, piezoresistive sensors, pressure sensor matrices, pressure visualizations, smart‐driving augmentations

## Abstract

Signal drift and hysteresis of flexible piezoresistive sensors pose significant challenges against the widespread applications in emerging fields such as electronic skin, wearable equipment for metaverse and human‐AI (artificial intelligence) interfaces. To address the creep and relaxation issues associated with pressure‐sensitive materials, a highly stable piezoresistive composite is proposed, using polyamide‐imide (PAI) fibers as the matrix and in situ grafted‐polymerized polyaniline (PANI) as the semi‐conducting layer. The PAI with large rigid fluorenylidene groups exhibits a high glass transition temperature of 372 °C (PAI 5‐5), which results in an extremely long relaxation time at room temperature and consequently offers outstanding anti‐creep/relaxation performances. The enhancement of PAI‐PANI interfacial bonding through in situ grafting improves the sensor reliably. The sensor presents high linear sensitivity of 35.3 kPa^−1^ over a pressure range of 0.2–20 kPa, outstanding repeatability, and excellent dynamic stability with only a 3.8% signal deviation through ≈10 000 cycles. Real‐time visualization of pressure distribution is realized by sensor matrices, which demonstrate the capability of tactile gesture recognition on both flat and curved surfaces. The recognition of sitting postures is achieved by two 12 × 12 matrices facilitated by machine learning, which prompts the potential for the augmentation of smart driving.

## Graphical abstract

A highly stable piezoresistive composite, based on polyamide‐imide (PAI) fibers and in situ grafted‐polyaniline (PANI), is employed to fabricate highly sensitive flexible pressure sensors with excellent repeatability and stability. Real‐time pressure distribution and tactile gesture recognition are enabled through sensor matrices. The integration of machine learning facilitates insightful applications such as driver posture recognition for augmentations of smart driving systems.

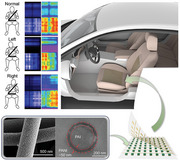

## 1 Introduction

Driver fatigue and distraction have consistently been significant causes of traffic accidents, culminating in substantial casualties and economic detriment.^[^ ^1^, ^2^ ^]^ The driver monitoring system, an essential intelligent apparatus, furnishes information pertaining to operational status of the driver, thereby serving as an efficacious method to augment the safety measures for both drivers and passengers.^[^ ^3^, ^4^, ^5^ ^]^ Predictive analysis of position and posture alterations induced by fatigue during vehicular operation can be conducted via pressure sensors deployed in driver's seat, thereby facilitating the detection of fatigue and somnolence.^[^ ^6^, ^7^, ^8^ ^]^ Consequently, a high‐resolution mapping of the pressure distribution on the seat and real‐time analysis on its variations are required to achieve higher posture recognition accuracy.

Pressure sensor matrices, an integration of multiple pressure sensors, have garnered special potential in such scenarios due to the capability of mapping and tracking pressure.^[^ ^9^, ^10^ ^]^ Various applications such as bionic hands,^[^ ^11^, ^12^ ^]^ smart medical equipment^[^ ^13^, ^14^, ^15^ ^]^ and tactile gloves^[^ ^16^, ^17^, ^18^, ^19^ ^]^ have raised high requirements for the conformability to curved surfaces with different curvatures. Significant efforts have been dedicated to broadening the pressure sensing regime,^[^ ^20^ ^]^ increasing the spatiotemporal resolution,^[^ ^21^ ^]^ and solving the mechanical crosstalk between pixels.^[^ ^22^, ^23^ ^]^ Nonetheless, it remains challenging to develop a pressure sensor matrix system that simultaneously satisfies flexibility, high sensitivity, good pixel uniformity, and more importantly, real‐time visualization of pressure distribution. These characteristics rely on the synergistic coordination of both software and hardware.

Flexible pressure sensors have demonstrated immense capability to function as sensing pixels of pressure sensor matrices by virtue of their fast response, simple fabrication, and compatibility with circuit printing. High‐performance flexible pressure sensors are in high demand as an essential component of robots equipped with artificial electronic skins, which have been developed to mimic human tactile sensation^[^ ^21^, ^24^, ^25^, ^26^, ^27^ ^]^ and facilitate efficient human–machine interaction.^[^ ^28^, ^29^ ^]^ Significant advancements have been achieved in flexible piezoresistive sensor to increase the sensitivity,^[^ ^30^, ^31^ ^]^ to extend the sensing range,^[^ ^30^, ^32^, ^33^ ^]^ to improve the detection limit,^[^ ^34^ ^]^ and to enhance the stability.^[^ ^35^, ^36^, ^37^ ^]^ Elastomeric materials, such as polyurethane (PU), polydimethylsiloxane (PDMS), Ecoflex, and hydrogels,^[^ ^38^, ^39^, ^40^, ^41^, ^42^, ^43^ ^]^ have been widely used in reported works. Such elastomeric polymers generally possess highly flexible molecular structures, including the backbone and side chains, which can easily move to dissipate the external force. This causes the material to creep under constant stress and to relax under sustained strain. The consequent plastic deformation and stress hysteresis inevitably lead to signal drift and serious hysteresis of the flexible sensor. Therefore, designing the polymer structure of the material to guarantee the anti‐creep/relaxation performance is crucial for the stable operation of the sensor.

In this work, a highly stable and recoverable flexible piezoresistive sensor is proposed, which is based on hot‐pressed polyamide‐imide (h‐PAI) fibers grafted with in situ polymerized semi‐conducting polyaniline (PANI). The issues of severe signal drift and low recoverability are improved by utilizing the anti‐creep and anti‐relaxation properties of specially designed PAI. The spatial hindrance of large rigid fluorenylidene units can significantly suppress the movement of polymer chains/segments, which is reflected by the elevated glass‐transition temperature *T*_g_ (390 °C) and the extremely long relaxation time. In general terms, brittleness comes along with high *T*_g_. Therefore, PAI is electrospun into thin films assembled from nanofibers to achieve great structural flexibility, which endows it with the capability to bend, twist, and conform to curved surfaces, as demonstrated in Figure S1 (Supporting Information). PANI is a biocompatible semiconducting polymer^[^ ^44^, ^45^, ^46^ ^]^ with high *T*_g_ (105 °C).^[^ ^47^ ^]^ Compared to other conducting polymers such as poly(3,4‐ethylenedioxythiophene) and polypyrrole, PANI has been reported to demonstrate lower cost, easier synthesis and processing, and higher environmental stability,^[^ ^48^, ^49^, ^50^ ^]^ which are significant merits for sensing applications. Therefore, PANI is in situ grafted from PAI with chemical interfacial bonding to achieve the PANI@PANI fibrous piezoresistive composites. Besides, it is feasible to form nanostructured PANI morphologies to enhance the physical friction against the relative movement of fibers under pressure loadings, which is beneficial for the improvement of sensor recoverability and signal stability.^[^ ^51^ ^]^ Compared to the sensors employing conventional elastomeric thermoplastic polyurethane (TPU), the PANI@PAI system displays much lower signal drift and far better recoverability. Hot pressing is employed to thermally joint‐weld PAI fibers, thereby impeding the relative movement between PAI fibers. The sensing stability is further enhanced based on h‐PAI. Employing the high‐performance PANI@(h‐PAI) sensors, the real‐time visualization of pressure distribution is demonstrated. To date, the imaging of pressure on plane surfaces has been widely studied and extensively demonstrated.^[^ ^20^, ^24^, ^52^, ^53^, ^54^ ^]^ However, various scenarios such as anthropomorphism hands,^[^ ^11^ ^]^ biomimetic skin^[^ ^55^ ^]^ and wind pressure measurement^[^ ^56^ ^]^ require the deployment of pressure sensor matrices on curved surfaces. This significantly amplifies the disparity in preloaded pressure among pixels, thereby reducing the pressure resolution of the sensor matrix. Consequently, the necessity of higher sensitivity and a broader pressure sensing range of each sensor pixel is raised. In addition, the algorithms for data processing and visualization require enhancement. The sensor matrix is capable of detecting the in‐plane movement of an object (1 g) on a flat surface. The application of pressure sensor matrix in this work is extended to curved surfaces. With effective compensation algorithms, the matrix system demonstrates the capability to recognize gestures such as single‐point touch, multi‐point touch and grasp. Furthermore, targeting the emerging smart driving realm, two 12 × 12 sensor matrices are deployed on a driver seat to recognize the sitting posture, which can be promising to assist the detection of driver drowsy and distraction, thereby facilitating the avoidance of severe traffic accidents.

## 2 Results and Discussion

**Figure 1:**
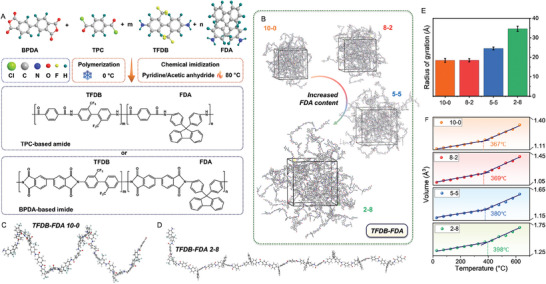
A) Design of PAI consisting of TPC‐based amide and BPDA‐based imide. B) Molecular dynamics (MD) models of PAI systems with varying TFDB‐FDA ratios (10‐0, 8‐2, 5‐5 and 2‐8). C,D) Molecular models of PAIs with different TFDB‐FDA ratios (10‐0 and 2‐8) built in MD simulations after relaxation. The increased content of FDA enhances the rigidity of the molecular chain. E) Radius of gyration corresponding to PAIs with varying FDA contents. F) Glass transition temperatures obtained from MD simulations.

Specially designed PAI employing rigid fluorenylidene groups is utilized as the matrix material by virtue of its outstanding anti‐creep properties and adjustable solubility. **Figure** **1**A illustrates the design and synthesis of PAI using terephthaloyl chloride (TPC) and 3,3′,4,4′‐biphenyltetracarboxylic dianhydride (BPDA) to form amide segments and imide segments, respectively. The trifluoromethyl groups in 2,2′‐bis(trifluoromethyl) benzidine (TFDB) can enhance the solubility of PAI in polar solvents. The addition of 4,4′‐(9‐fluorenylidene) dianiline (FDA), which features in the large rigid fluorenylidene unit, can suppress the movement of the backbone and segments via increasing the spatial hindrance. Accordingly, the anti‐creep and anti‐relaxation performances can be expected to achieve further improvement. Molecular dynamics (MD) simulations are conducted to investigate how the incorporation of FDA affects the structural properties of the polymer chain. As illustrated in Figure 1B–D and Figure S2 (Supporting Information), the increased engagement of FDA significantly reduces the winding of the polymer chain. This subsequently results in the decrease in the radius of gyration, as presented in Figure 1E. This chain rigidification effect will help to prohibit the energy dissipation via molecular chain movement when creep/relaxation is initiated. Consequently, the increased spatial hindrance and chain rigidity work in tandem and result in the elevation of *T*_g_, as shown in Figure 1F.

**Figure 2:**
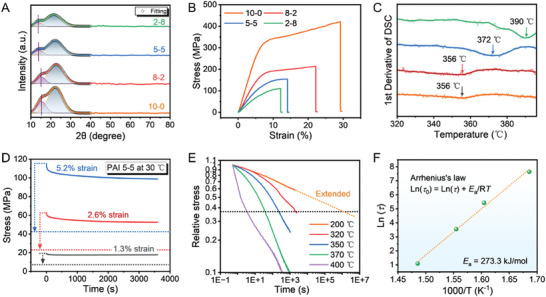
A) X‐ray diffraction patterns of PAI. B) Impacts of the TFDB‐FDA ratio upon the mechanical properties of PAI. C) Glass transition temperatures of PAI obtained from the first derivative of DSC curves. D) Stress relaxation tests of PAI 5‐5 performed at 1.3%, 2.6%, and 5.2% strain and the corresponding reference lines for 63.2% (corresponding to 1‐1/e) relaxation. E) Normalized stress–relaxation curves of PAI 5‐5 at different temperatures. F) Relaxation times of PAI 5‐5 at varied temperatures, which agree with Arrhenius’ law.

PAIs with varied TFDB‐FDA ratios are synthesized by chemical imidization. Figure S3a,b (Supporting Information) shows the FTIR spectra of as‐synthesized PAIs. The detailed peak assignments are listed in Table S1 (Supporting Information). The appearance of new peaks at 1509 cm^−1^ exclusively in FDA‐contained PAIs indicates the successful incorporation of FDA. Besides, the decrease of TFDB content causes the blueshift of the C═O peaks in the imide ring, which includes the asymmetric stretch at ≈1777 cm^−1^ and the symmetric stretch at ≈1722 cm^−1^, as presented in Figure S3c (Supporting Information). The redshift originates from the diminished weakening effect of ─CF_3_ groups in TFDB upon the inductive effect of nitrogen atoms in the imide ring. To explore the impacts of the TFDB‐FDA ratio upon the crystallinity properties of PAI, X‐ray diffraction (XRD) patterns are measured and given in **Figure** **2**A. After peak fitting, two amorphous peaks can be observed. The purple peak at around ≈15° corresponds to the intermolecular packing originating from the high polarity of ─CF_3_ groups. As the content of FDA increases, its large rigid fluorenylidene groups inhibit such intermolecular packing,^[^ ^57^ ^]^ resulting in the intensity decay of the purple peak. In the meanwhile, the angle value also exhibits a trend of decrease (15.3°, 15.1°, 13.8° and 13.6° corresponding to 10‐0, 8‐2, 5‐5 and 2‐8), indicating an expansion of the mean intermolecular distance (d‐spacing).

The mechanical properties with dependence on the TFDB‐FDA ratio are also investigated, as displayed in Figure 2B and Figure S4 (Supporting Information). The tensile modulus, which is essentially related to the reversible stretching of chemical bonds, reflects the rigidity of the molecular chain backbone. After the incorporation of FDA, the tensile modulus of PAI declines from 5.53 to 1.53 GPa, as the quaternary carbon in FDA is more stretchable than the biphenyl unit in TFDB. Differential scanning calorimetric (DSC) curves of as synthesized PAIs are shown in Figure S5 (Supporting Information). The glass transition temperature (*T*_g_) is determined via calculating the 1^st^ derivative of the heat flow with respect to temperature, as shown in Figure 2C. As the FDA content increases from 0 to 80%, the *T*_g_ of PAI rises from 356 to 390 °C. This is attributed to the enhanced spatial hindrance against chain movement caused by the large rigid structure of fluorenylidene in FDA.^[^ ^58^ ^]^ This is consistent with the positive correlation between *T*_g_ and FDA content obtained by MD simulations (Figure 1F). Minor discrepancies between numerical and experimental results are attributed to the smaller chain number and higher cooling rate employed in the MD simulations. Featuring in the high *T*_g_, the relaxation and creep behaviors of the as‐designed PAI can be suppressed even at high temperature.

The fabrication procedure of fibrous PANI@PAI composites commences with the electrospinning of PAI. Although PAI 2–8 demonstrates the highest *T*_g_ guaranteeing the best anti‐creep performance, it is not suitable for electrospinning due to the high viscosity of the precursor solution (even at a low concentration of 5 wt%). Therefore, PAI 5‐5 is selected for the subsequent fabrication procedure. Correspondingly, stress‐relaxation experiments are performed to investigate the anti‐relaxation properties of PAI 5‐5. The relaxation time (*τ*) is defined as the duration required for the stress to relax to 36.8% (1/e) of its initial value, which reflects the resistance against segment movement and chain rearrangement.^[^ ^59^ ^]^ As shown in Figure 2D, the stress of PAI 5‐5 reaches stabilization with a relaxation rate of ≈10% under different strains (1.3%, 2.6% and 5.2%), indicating an extremely long relaxation time at room temperature (30 °C). Normalized stress–relaxation curves of PAI 5‐5 at different temperatures around its *T*_g_ are presented in Figure 2E. PAI 5‐5 demonstrates an extraordinarily long relaxation time of approximately 1E6 seconds even at 200 °C, which indicates the outstanding anti‐relaxation properties. According to Arrhenius’ law, the activation energy (*E*_a_) required to initialize the relaxation is calculated to be 273.3 kJ mol^−1^ (Figure 2F), which is much higher than elastomeric TPU (≈45 kJ mol^−1[^ ^60^ ^]^), waterborne polyurethane (≈26.2 kJ mol^−1[^ ^61^ ^]^), PDMS (≈88.5 kJ mol^−1[^ ^62^ ^]^) and styrene butadiene rubber (≈80 kJ mol^−1[^ ^63^ ^]^). This suggests the ample resistance against segment movement and chain rearrangement, guaranteeing the excellent anti‐relaxation and anti‐creep performances of PAI 5‐5. Accordingly, PAI 5‐5 demonstrates a lower creep compliance (≈0.35 GPa^−1^, refer to Figure S6, Supporting Information) than nanoparticle reinforced polyimide.^[^ ^64^ ^]^ Interestingly, the temporal dependence of the creep compliance exhibits a trend of gradual decrease after it reaches ≈0.35 GPa^−1^. This has been further verified by molecular dynamics simulations in Figure S7 (Supporting Information). The external stress causes an increase in the orientation of PAI molecular chains, leading to the increase of the conformational entropy. The generated entropic stress consequently causes the contraction of polymer chains.^[^ ^65^, ^66^ ^]^ Therefore, this endows PAI 5‐5 with the capability to recover from creep‐induced plastic deformation, which is beneficial to the long‐term stability.

**Figure 3:**
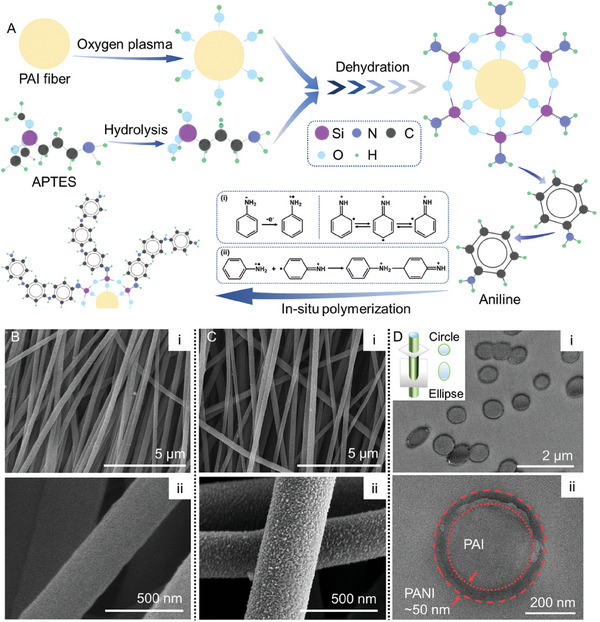
Fabrication and characterizations of PANI@PAI. A) Schematic of the preparation procedure via in‐situ polymerization including three steps: hydrophilization using UV‐ozone treatment, surface functionalization with ─NH_2_ terminals using APTES, and in situ grafted‐polymerization of PANI. B) Scanning electron microscope (SEM) images of pristine PAI fibers. C) SEM images of PANI@PAI fibers, showing the nanobumps of PANI. D) Transmission electron microscope (TEM) images of PANI@PAI fibers, showing the thickness of the PANI layer.

**Figure** **3**A elucidates the grafting mechanism of PANI from PAI fibers. The ultraviolet‐ozone treatment induces active ─OH groups on the surface of PAI fibers. The ─Si─OH groups generated from the hydrolysis of (3‐aminopropyl) triethoxysilane (APTES), chemically bond with ─OH groups on the surface of PAI fibers and simultaneously form cross‐linked ─Si─O─ networks through inter‐bonding. Consequently, the outmost surface is functionalized with terminal ─NH_2_ groups, which serve as the grafting sites for the polymerization of PANI. Specifically, ─NH_2_ group attacks the active 4′‐carbon of aniline molecules (or oligomers) induced by electron resonance.^[^ ^67^, ^68^, ^69^ ^]^ Under the oxidization of ammonia persulphate (APS), chains of aniline oligomers will extend. Concurrently, H^+^ ionized from citric acid (CA) coordinates with ─N─ in PANI, creating holes as electrical carriers.

**Figure 4:**
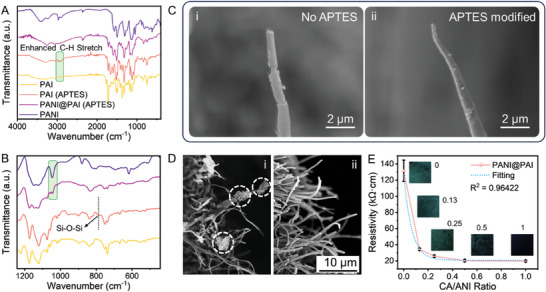
Effects of APTES in the in situ grafting of PANI. A,B) FTIR spectra of PAI fibers, APTES‐modified PAI fibers, PANI‐grafted APTES‐modified PAI fibers and PANI powder. C) SEM images showing the breaking end of stretched PANI@PAI fibers without (i) and with (ii) APTES modification. D) SEM images showing the morphologies of PANI@PAI fibers without (i) and with (ii) APTES modification. E) Impacts of the CA/ANI ratio upon the electric resistivity of PANI@PAI, displaying a trend of exponential decay due to the increased doping level of PANI.

Figure 3B,C is the SEM images of pristine PAI fibers and PANI@PAI fibers, demonstrating the uniformity of the fiber diameter. The magnified view of the PANI@PAI fibers reveals the nanostructured morphology of the PANI coating, which helps to hinder the relative movement of fibers via physical friction. Besides, PANI residuals can hardly be observed, indicating the controllability of the fabrication procedure, which is advantageous for the stability and repeatability of manufacturing quality. Figure 3D presents the TEM image of the cross‐section of PANI@PAI fibers. Figure 3D–I shows the uniform distribution of the diameter of PANI@PAI fibers. The shape of the cross‐section (circle or ellipse) is determined by the fiber orientation, as demonstrated in the inset. A magnified view of a representative fiber in Figure 3D‐ii presents three zones with different color contrasts. The inner circle and the outmost surrounding (light gray) are the PAI fiber and the epoxy matrix, respectively. The dark gray ring corresponds to the PANI coating with a thickness of ≈50 nm. **Figure** **4**A provides the FTIR spectra of pristine PAI fibers, PANI (doped with CA) powders, and PANI@PAI fibers. Detailed assignment of characteristic peaks is listed in Table S2 (Supporting Information). The enhanced C─H stretch peak at ≈3000 cm^−1^ and the appearance of Si─O─Si peak at ≈790 cm^−1^ confirm the successful surface modification illustrated in Figure 3A. The appearance of the characteristic peak of PANI (1055 cm^−1^) in the spectrum of PANI@PAI evidently confirms the successful grafting of PANI, as shown in Figure 4B. To further validate the effectiveness of in situ grafting in enhancing interfacial strength, PANI@PAI fibers with and without APTES modification are stretched until breaking. The SEM images in Figure 4C present the evident interface failure of PANI@PAI fiber without APTES modification at the breaking end. The PANI coating detaches from the PAI core at multiple sites. In contrast, by bridging the formation of chemical bonding between PAI and PANI, APTES significantly improves interface robustness, which guarantees the durability of pressure sensors. Furthermore, APTES modification can also promote the uniform growth of PANI around PAI fibers, which is beneficial for the reproducibility of the material. By contrast, amorphous PANI residuals can be observed around PAI fibers without APTES modification, as shown in Figure 4D. The dependence of the electrical resistivity upon the doping content of CA is presented in Figure 4E, which displays a trend of exponential decay as the CA/ANI ratio increases, along with the darkening of the greenish color.

**Figure 5:**
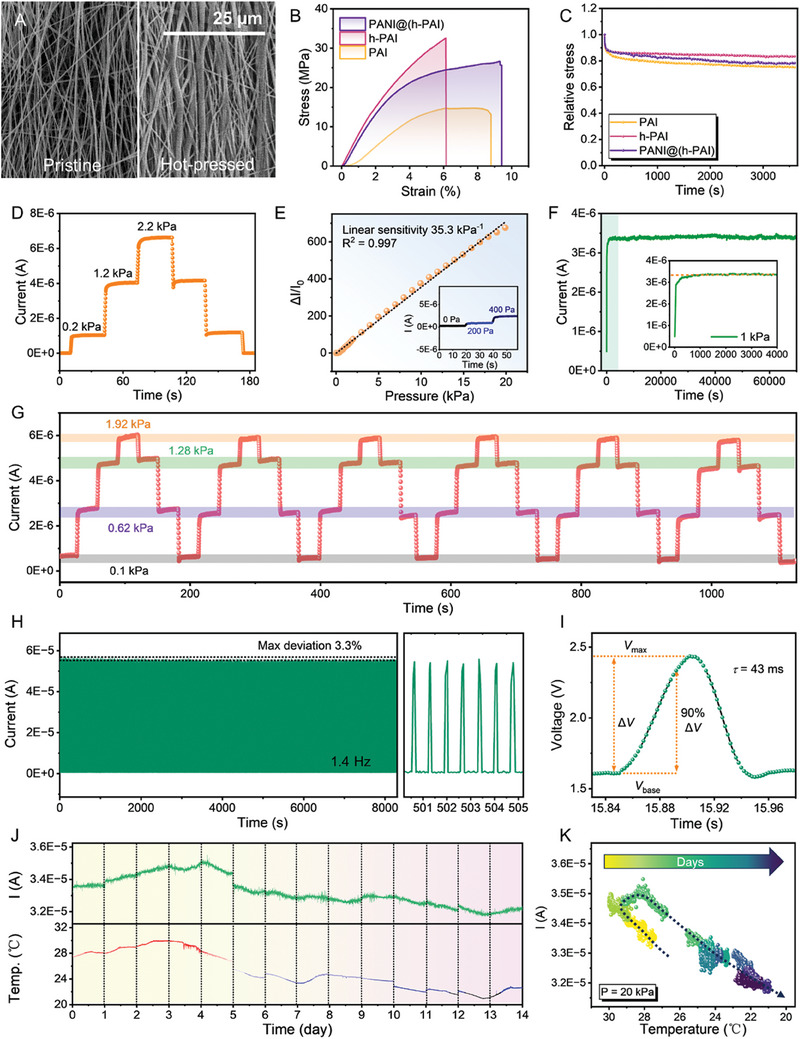
Pressure‐sensing performances of PANI@(h‐PAI) fibrous composites and the corresponding pressure sensors. A) SEM images of PANI@PAI and PANI@(h‐PAI) fabricated from pristine PAI mats and hot‐pressed mats. B) Strain–stress curves and C) stress relaxation curves of pristine PAI, h‐PAI and PANI@(h‐PAI). D) Step loading test of the sensor based on PANI@(h‐PAI).). E) Relative change of the current signal under different pressure loadings, indicating the good linear sensitivity (35.3 kPa^−1^) of PANI@(h‐PAI) across a broad pressure range of 0.2–20 kPa. F) Current signal recorded continuously for ≈24 h under a constant stationary pressure of 1 kPa, showing the great stability of PANI@(h‐PAI). G) Multiple step loading–unloading cycles, with a baseline pressure of 0.1 kPa. H) ≈10 000 cycles of pressure loading and unloading at 1.4 Hz. The flat outlines of the base signal and the response signal demonstrate the superb durability and dynamic stability of the sensor. I) Response time of the pressure sensor. J,K) Long‐term temperature dependence of the sensor signal through 14 d under a pressure loading of 20 kPa.

The pressure‐sensing performances of PANI@PAI fibrous composites are evaluated. Step loading and unloading tests are conducted to investigate the reversibility of PANI@PAI as a pressure‐sensitive material. Compared to conventional elastomer‐based PANI@TPU, the consistency between the loading signal and the unloading signal indicates the excellent stability and recoverability of PANI@PAI, as shown in Figure S8 (Supporting Information). However, slight signal drift can still be observed at 1.6 kPa and 2.8 kPa, which is assumed to be caused by the movement of fibers. To accelerate the stabilization of the signal, PAI fibrous mats are pre‐treated with hot‐pressing at a high temperature of 370 °C (near *T*_g_), with a high pressure loading of 500 kPa for 2 h, before the in situ grafting of PANI. It is expected that PAI fibers will be thermally joint‐welded to form a crosslinking network. SEM images in **Figure** **5**A show that the fibers tend to pile compactly and orderly after the hot‐pressing treatment. Moreover, fiber bundles in hot‐pressed PAI (denoted as h‐PAI) are formed from several adjacent fibers under the synergism of the high temperature and pressure. The stress–strain curves in Figure 5B demonstrate that h‐PAI owns higher modulus than pristine PAI, which can be attributed to the increased inter‐fiber compactness. As for PANI@(h‐PAI), it can be regarded as a core–shell composite, as depicted in Figure 3C,D. Its elastic modulus can be expressed by the rule of mixture for core–shell fibers as follows:

(1) $$E_{\mathrm{PANI}@\left(h-\mathrm{PAI}\right)}=E_{\mathrm{PAI}}A_{\mathrm{PAI}}+E_{\mathrm{PANI}}A_{\mathrm{PANI}}$$

(2) $$A_{\mathrm{PAI}}+A_{\mathrm{PANI}}=1$$

where *E* and *A* represent the modulus and cross‐sectional area ratio, respectively. Therefore, PANI@(h‐PAI) shows a lower modulus than h‐PAI as *E*_PANI_ (0.9 GPa^[^ ^70^ ^]^) is lower than *E*_PAI_ (2.3 GPa, PAI 5‐5). Moreover, stress relaxation tests are also carried to investigate the effects of hot‐pressing in improving the mechanical stability of fibrous composites, as shown in Figure 5C. In this scenario, the stress relaxation originates from not only the molecular‐scale movement polymer chains, but also the micro‐scale movement of fibers. Accordingly, h‐PAI exhibits lower stress relaxation than pristine PAI because the fiber movement is suppressed due to inter‐fiber crosslink resulting from the hot‐pressing induced welding effect. The grafting of PANI slightly exacerbates the stress relaxation because its creep resistance is significantly inferior to that of PAI, as can be inferred from their *T*gs. Still, PANI@(h‐PAI) shows lower stress relaxation than pristine PAI. This can be partially attributed to the increased friction resistance against fiber movement due to the rough nanostructured morphology of PANI modification.

Then pressure sensors are assembled with encapsulation as depicted in Figure S9 (Supporting Information). To avoid the impacts of environmental humidity and oxygen upon the electrical conductivity of PANI, a ring‐shaped tape is employed to ensure that air cannot get in touch with PANI@(h‐PAI). This can effectively improve the environmental durability of the sensor. The voltage sweeping curves in Figure S10 (Supporting Information) indicate the good linear ohmic properties of PANI@(h‐PAI) when the applied pressure increases from 0 to 4 kPa, showing the reliability of the conductive networks over a broad pressure range, which is beneficial for the stable performances of the pressure sensor. As shown in Figure 5D, the stability of PANI@(h‐PAI) is greatly enhanced. The current signal stabilizes more quickly than that of the sensor based on untreated PAI. The sensitivity (Figure 5E) is calculated from the linear fitting of the relative change of the current signal (Δ*I*/*I*_0_, *I*_0_ = 4.83 × 10^−8^ A) corresponding to varied loading pressure, which is calibrated utilizing a commercial force gauge (Figure S11, Supporting Information). PANI@(h‐PAI) displays high linear sensitivity of 35.3 kPa^−1^ across a broad pressure range of 0.2–20 kPa. This high linear sensitivity is attributed to the formation of multiple conducting paths when numerous fibers contact each other under applied pressure. Figure 5F presents the stationary stability of PANI@(h‐PAI), which is evaluated at a constant pressure loading of 1 kPa for more than 24 h. Figure 5G demonstrates the dynamic recoverability and repeatability of the pressure sensor. The average current signals corresponding to the loading steps and unloading steps exhibit great consistency. The dynamic stability is evaluated with ≈10 000 loading–unloading cycles (15 kPa). The stable profiles of both the baseline signal and the response signal demonstrate the outstanding reliability and repeatability, as displayed in Figure 5H. Due to the limitation of the sampling rate of Keithley 2636B, the response time of the pressure sensor is measured using a professional data acquisition card (NI USB‐6008), according to the measurement setup in Figure S12 (Supporting Information). The response time here corresponds to the time interval for the sensor signal to reach 90% of the maximum once the external pressure is applied, as shown in Figure 5I. The sensor demonstrates a swift response time of 43 ms. **Table** **1** lists the characteristics of some previously reported sensors with excellent performance. Evidently, the proposed sensor based on high‐*T*g PANI@(h‐PAI) with in situ grafting interface excels in linear sensitivity, sensor stability and signal drift compared to the most widely reported high‐performance elastomeric systems.

**Table 1:** Comparison of sensing performances with typical pressure sensors based on elastomeric systems (N.A.: not available).

Material	Sensitivity [kPa⁻¹]	Sensing range [kPa]	Response time [ms]	Cyclic stability (cycles)	Signal drift [%]	Refs.
Ultrathin nanofiber‐based PDMS sprayed with CNT	92.11 (0–0.1 kPa) 4.93 (0.1–1.5 kPa)	0–1.5	85 ms	1000	N. A	[71]
Microstructured PDMS coated with PEDOT:PSS	2.32	0–100	240 ms	2000	≈33%	[40]
Porous CNT/CB/CIP/silicone composites	0.304	0–150	88 ms	≈4500	≈37%	[72]
Gaussian‐shaped CNT/PDMS composites	1.77	0.02–30	25 ms	10000	≈8%	[73]
Silicone sprayed with rG	133 (0–40 kPa) 34.4 (40–100 kPa) 11.4 (100–300 kPa)	0–300	25 ms	10000	≈17%	[74]
MXene/NH_2_‐CNTs/ melamine sponge	1.03 (0–5 kPa) 10.8 (5–12 kPa) 1.01 (12–30 kPa)	0–30	40	5000	≈15%	[75]
3D‐printed PDMS coated with PEDOT: PSS	160 (0–0.577 kPa) 0.16 (10–28 kPa)	0–28	114	4000	≈5%	[76]
MXene/PPy@PDMS sponge	6.9 (0–15 kPa) 0.02 (70–300 kPa)	0–300	100	5000	N.A.	[77]
Microdome‐structured PDMS/CNTs/PPNWFs	6.31 (0–50 kPa) 3.07 (50–800 kPa)	0–800	72 ms	10000	≈14%	[78]

To investigate the inevitable temperature drift of the semiconductor‐based sensor, the amplitude of the current signal across a broad temperature range (‐70–50 °C) is recorded to lay the foundation of temperature compensation, as shown in Figure S13 (Supporting Information). The signal presents a surprising non‐exponential dependence upon the Kelvin temperature, which is inherently attributed to the conducting mechanism of PANI. At low temperatures, PANI exhibits semiconductor characteristics. The conducting mechanism is dominated by thermal activation, which can be described with an Arrhenius equation. While at higher temperatures, it behaves metallic and the electrical conductivity decreases owing to the dominance of de‐protonation/de‐doping over thermal activation.^[^ ^79^ ^]^ As a result, the environmental temperature could be an inevitable factor that affects the stability of the sensor, since the electrical conductivity of PANI semi‐conductor demonstrates significant dependance upon the temperature. Therefore, the pressure sensor is placed under ambient environment for 14 days, with a loading pressure of 20 kPa, to thoroughly investigate its long‐term performance. The room temperature *T* and the sensor signal *I* are synchronously recorded. As presented in Figure 5J, *T* and *I* display similar trends with respect to the time *t*. The *I*–*T–t* dependence is subtracted and plotted as a color mapping in Figure 5K. An increase in the amplitude of signal *I* at ≈28 °C can be observed after long‐term high‐pressure loading, which could be caused by the plastic deformation of PANI since its *T*g is much lower than PAI. Overall, *I* is positively correlated to *T*. After linear fitting, temperature compensation is implemented, which effectively decreases the temperature‐induced deviation by ≈50% (Figure S14, Supporting Information). Besides, the static stability is also investigated under different temperatures to characterize its thermal durability. Signal degradation could only be observed when the temperature exceeds 60 °C, which demonstrates the good thermal stability of the sensor (Figure S15, Supporting Information).

**Figure 6:**
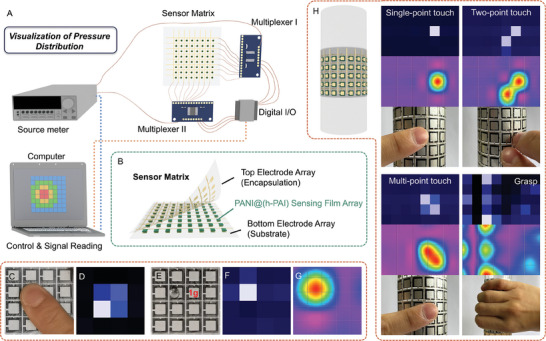
A) The system setup of pressure visualization, including the source meter, sensor matrix, digital I/O, two multiplexers, and a computer for system control, signal reading, and data recording. B) Structure of the sensor matrix. C,D) Pressure distribution directly transduced from the current amplitude of each pixel. High pressure is required to distinguish the free/pressed state of each pixel. E,F) Pressure distribution after baseline compensation. The signal is transduced from the relative change of the current amplitude. G) Pixelated colormap visualizing the distribution of pressure via interpolation algorithm. H) Illustration of pressure sensor matrix working on a convex surface and pressure mapping of gestures including single‐point touch, two‐point touch, multi‐point touch, and grasping.

The sensor matrix for the real‐time visualization of pressure distribution is constructed with a bottom electrode array on a PET substrate, a sensing film matrix, and a top electrode array on a PET encapsulation film. The system setup is illustrated in **Figure** **6**A,B. The sensor matrix can recognize finger touch using the current amplitude of each pixel as the indication of pressure intensity, as demonstrated in Figure 6C,D. To improve the pressure sensitivity and spatial resolution, a baseline compensation algorithm is employed to characterize the relative variation of the current amplitude at each pixel. The initial current amplitude of a 4×4 matrix is acquired for 5 times and stored in a 4×4×5 dataset. The average current amplitude of each pixel is calculated (4×4 dataset), and set as the baseline to accomplish the system initialization. As demonstrated in Figure 6E,F, the sensor matrix can distinguish the pressure loading of 1 g (≈100 Pa) and sense its movement (Figure S16, Supporting Information). Additionally, a 2D bilinear interpolation algorithm is implemented to smooth the graphical visualization of pressure distribution, as shown in Figure 6G.

**Figure 7:**
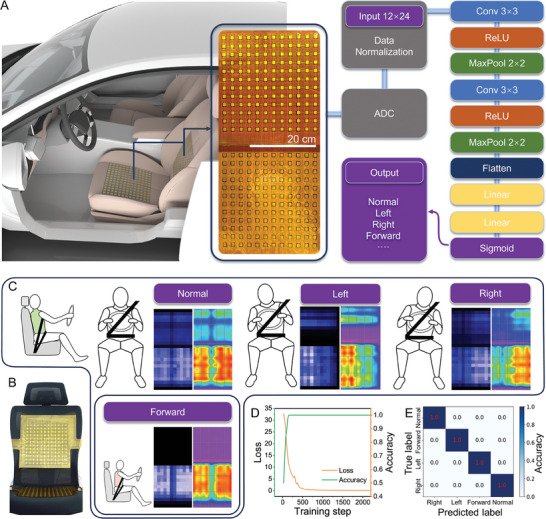
A) Schematic of the smart sitting posture recognition system facilitated by pressure sensor matrices and machine learning. The output of the neural network gives the classification of sitting postures including normal, left, right, and forward. B) Photograph of pressure sensor matrices deployed on the base and backrest of the seat. C) Pressure mapping of the sensor matrices corresponding to different sitting postures. D) Training loss and accuracy of the CNN model as the training step increases. E) Normalized confusion map of the neural network.

To explore the feasibility of the sensor matrix on curved surfaces, an 8 × 5 matrix is attached to a convex surface with a curvature radius of 8 cm, as shown in Figure 5H. It presents the capability of gesture recognition such as single‐point touch, multi‐point touch, and grasp. Furthermore, the promising application scenario of the sensor matrix, by virtue of its conformability and high sensitivity, has been extended to the recognition of sitting postures to facilitate the intelligence of smart driving systems. As aforementioned, this can assist in the prompt detection of fatigued driving and distracted driving, thereby decreasing the occurrence of severe traffic accidents. As illustrated in **Figure** **7**A,B, two pressure sensor matrices (12 × 12) are deployed on the seat base and the backrest, respectively. The data acquired by ADC, which is a 12 × 24 array, is normalized and input into a convolutional neural network (CNN). The data features are extracted through the convolutional layer and filtered through the ReLU layer. Finally, the sigmoid layer outputs the classification result of the sitting posture, including normal, left, right, and forward (Figure 7C). Employing the cross‐entropy loss and SGD optimizer, the accuracy of the CNN model increases as the iterative training step increases, as depicted in Figure 7D. Figure 7E presents the classification confusion matrix of the CNN model, which indicates a recognition accuracy of 100%. This demonstrates the capability of the pressure sensor matrices in sitting posture recognition, which can be promising to augment the hardware of smart driving systems.

## 3 Conclusion

In conclusion, a highly stable and recoverable flexible pressure sensor based on specially designed PAI fibers with in situ grafted PANI is developed. The incorporation of rigid fluorenylidene groups elevates the *T*_g_ of PAI up to 390 °C, indicating a significant improvement in anti‐creep and anti‐relaxation performances. Furthermore, the restraining of PAI fiber movement induced by the joint‐welding effect of a special hot‐pressing treatment, greatly enhances the pressure‐sensing stability. The sensor displays impressive repeatability and recoverability, high linear sensitivity of 35.3 kPa^−1^ (0.2–20 kPa) and a quick response time of 43 ms, indicating its potential for both dynamic and stationary pressure monitoring. Pressure sensor matrices are assembled with a customized setup of data acquisition and processing. The baseline compensation improves the sensitivity (100 Pa) of the pressure sensor matrix, enabling object movement tracking and gesture recognition on planar and curved surfaces. The sensor matrices demonstrate the potential to facilitate the detection of driver's sitting postures in smart driving. To look beyond, pressure sensor matrices can be deployed on the steering wheel to provide another dimension of infotainment system control, or to assist the detection of driver drowsy by detecting the gripping force of the driver. It is also anticipated that this high‐performance pressure sensor and the demonstrated sensor matrix can prompt the applications of flexible pressure sensors in bionic robots, trajectory tracking, and advanced intelligent driving systems.

## 4 Experimental Section

### Materials

2,2′‐Bis(trifluoromethyl) benzidine (TFDB), 3,3′,4,4′‐biphenyltetracarboxylic dianhydride (BPDA), terephthaloyl chloride (TPC), pyridine (Py), N,N‐dimethylpyridin‐4‐amine (DMAP), aniline and ammonia persulphate (APS) were purchased from MACKLIN. 4,4′‐(9‐Fluorenylidene) dianiline (FDA) was purchased from Bidepharm. Acetic anhydride, dimethylacetamide (DMAC), N‐methylpyrrolidone (NMP), N, N‐dimethylformamide (DMF), and tetrahydrofuran (THF) were purchased from Shanghai HUSHI. Trimethylchlorosilane (TMSCl), (3‐aminopropyl) triethoxysilane (APTES), and citric acid (CA) were bought from Aladdin.

### Synthesis of Polyamide‐Imide

TPC and BPDA (1/1 mol/mol) were used to construct the amide segment and imide segment of polyamide‐imide, respectively. In a typical procedure, TFDB and FDA (20 mmol in total) were dissolved in 60 mL of DMAC under the protection of nitrogen atmosphere in a three‐neck flask (250 mL). The temperature was cooled down to 0 °C and 3.054 g of TMSCl (20 mmol), 0.244 g of DMAP (2 mmol), and 2.25 mL of pyridine were added to the solution and stirred for 30 min. Then BPDA and TPC (20 mmol in total) were added and the mixture was kept stirring at 0 °C for 3 h and at room temperature for 12 h. The polymer solution was heated to 80 °C, and 4.5 mL of acetic anhydride and 3.5 mL of pyridine were injected to initiate the chemical imidization. After 2 h, PAI was precipitated with EtOH/H_2_O (1/1 by volume) and washed twice under ultrasonication for 90 min, and dried in oven at 60 °C for 10 h.

### Electrospinning of PAI

PAI (5‐5) was dissolved in a ternary solvent consisting of 13 mL of NMP, 3 mL of DMF, and 5 mL of THF. The solution was transferred into a syringe with a 21 G needle. The electrospinning was carried out using a YFSP‐T machine from Tianjin Yunfan Tech for 6 h (voltage: 21 kV, needle‐to‐collector distance: 15 cm, collector rotation speed: 300 rpm, feed rate: 5.4 mL h^−1^). The electrospun PAI mats were dried in oven at 60 °C for 5 h and annealed at 200 °C for 2 h.

### Preparation of PANI‐Grafted PAI

A sheet of electrospun PAI mat (2 cm × 2 cm) was immersed in an APTES/ethanol (1/10 by volume) for 1 h and then rinsed three times in deionized water. The PAI sheet was soaked in 15 mL of aqueous solution of ANI (0.2 mmol/mL) and CA (0.05 mmol/mL), and stored in fridge for 1 h. Then 15 mL of APS solution (0.2 mmol mL^−1^, pre‐cooled with ice bath) was added to trigger the oxidization of ANI. The reaction was sustained in fridge for 12 h to accomplish the grafting and polymerization of PANI. The PANI‐grafted PAI mat was cleansed with deionized water under ultrasonication for 20 s. The procedure was repeated for three times. The mat was dried in oven at 60 °C for 10 h.

### Fabrication of Pressure Sensor Based on PANI‐Grafted PAI

Customized interdigital electrodes were screen‐printed on a PET substrate (0.03 mm) using silver paste. A thinner PET film (0.005 mm) was applied to encapsulate a circular PANI‐grafted PAI on the electrodes using thermoset adhesive, in a glove chamber with nitrogen atmosphere.

### Characterizations

The relative molecular weight of PAI 5‐5 (*M*w = 173320, PDI = 1.496) was measured employing gel permeation chromatography (GPC, Waters 1515), as displayed in Figure S17 (Supporting Information). The thermal stability of PAI 5‐5 was evaluated using thermogravimetry (Mettler Toledo, TGA/DSC3+). The decomposition temperature Td5% was 480 °C, as shown in Figure S18 (Supporting Information). X‐ray diffraction (XRD) patterns were carried out to determine the crystallinity of PAI after annealing using Shimadzu 7000 X‐ray diffractometer (Cu K_α_, *λ* = 1.5406 Å), with a scanning range of 10°–80°. Differential scanning calorimetric (DSC) curves were measured to determine the *T*_g_ of PAI using TA Q200 (TA Instruments). Strain‐stress analysis was carried on a tensile test machine to analyze the impacts of TFDB/FDA composition on the mechanical properties of PAI. Scanning electron microscopic (SEM) images were taken using Hitachi S4800. Fourier transform infrared (FTIR) spectra (400–4000 cm^−1^) were measured using Nicolet 5700 FTIR spectrometer in total reflection (ATR) mode, to verify the grafting of PANI on PAI. Electrical resistivity was measured with a standard four‐probe method. Electrical conductivity was calculated from the resistivity according to the equation (*σ* = 1/*ρ*).

### Pressure‐Sensing Performance of PANI@PAI Mats

The voltage sweep curves were acquired with Keithley 2636B controlled using a Python script. The step loading–unloading tests were carried out on a homemade stage with fixtures for the PANI@PAI fibrous mats. The pressure was altered by adding or removing standard weights (1 and 5 g). The sensitivity curve was obtained utilizing a commercial force gauge, as illustrated in Figure S11 (Supporting Information).

### Setup of Pressure Sensor Matrix

The pressure sensor matrix consists of a bottom electrode array, a top electrode array and a sensing film array. Detailed geometric parameters were given in Figure S19 (Supporting Information). The intersectional points on the bottom electrode array were screen printed with insulating paste to avoid short‐circuit circumstances. Thermoset adhesive was employed to complete the assembling of the sensor matrix. The sensor matrix was connected to two CD74HC4067 multiplexers from Texas Instruments. The switching of current channel was controlled utilizing the digital output channels of a data acquisition card (NI USB 6008). The current measurement was performed using a source meter (Keithley 2636B). The plane sensor matrix system was controlled by a LabView program, and the cylindrical system was controlled via a python program using official APIs (nidaqmx and pyvisa) to communicate with NI devices.

### Molecular Dynamics Simulations

PAI systems with different proportions of TFDB and FDA were subjected to molecular dynamics (MD) simulations to predict their glass transition temperatures (*T*_g_s). The initial molecular model was constructed using Materials Studio software, and MD simulations were performed with LAMMPS, employing the polymer consistent force field (PCFF), which is suited for synthetic polymers due to its parameterization for a broad range of organic molecules. Temperature and pressure control were achieved through the Nose‐Hoover thermostat and barostat, respectively. Periodic boundary conditions were applied to reduce surface effects. The repeat unit ratios of TFDB and FDA for systems 1# through 4# were 10: 0, 8: 2, 5: 5, and 2: 8, respectively. Each system underwent a relaxation process for 500 ps in the NVT ensemble followed by 1 ns in the NPT ensemble at 900 K. After equilibration, each system was cooled to 300 K with a cooling rate of 50 K/500 ps, and their *T*_g_ values were evaluated.

## Conflict of Interest

The authors declare no conflict of interest.

## Author Contributions

K.C. and H.Y. contributed equally to this work. K.C. designed and conducted the experiments, analyzed the data, and composed the manuscript. H.Y. and A.W. performed the molecular dynamics simulations. All authors revised the manuscript.

## Supporting information



Supporting Information

## Data Availability

The data that support the findings of this study are available from the corresponding author upon reasonable request.
