# Supplementary material for: Smart Driving Hardware Augmentation by Flexible Piezoresistive Sensor Matrices with Grafted‐on Anticreep Composites
Source: Adv Sci (Weinh). 2024 Nov 25;12(3):2408313. doi: 10.1002/advs.202408313 (PMC11744520; doi:10.1002/advs.202408313)
Supplement: Supplementary file 1 — Supporting Information [file ADVS-12-2408313-s001.docx]

Supporting Information

**Smart driving hardware augmentation by flexible piezoresistive sensor matrices with grafted-on anti-creep composites**

*Kaifeng Chen^#^, Hua Yang^#^, Ang Wang, Linsen Tang, Xin Zha, Ndeutala Selma Iita, Hong Zhang, Zhuoxuan Li, Xinyu Wang, Wei Yang, Shaoxing Qu, Zongrong Wang**


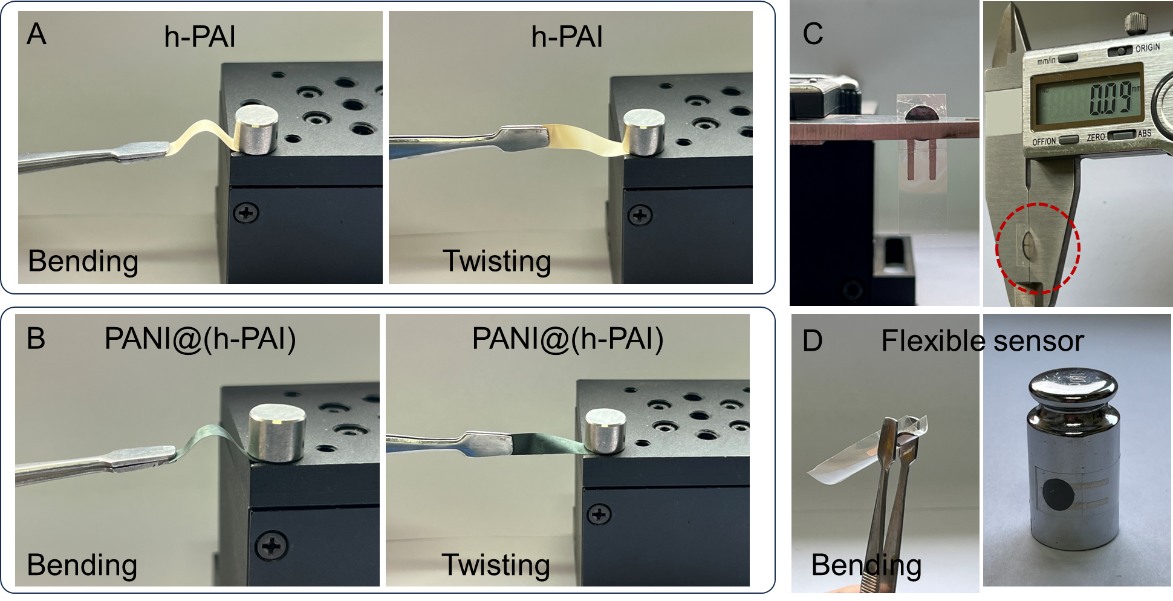


**Figure S1.** a-b) Flexibility of h-PAI and PANI@(h-PAI), which enables them to bend and twist. c) The thickness of the flexible pressure sensor based on PANI@(h-PAI). d) Bending capability and conformability of the flexible pressure sensor.


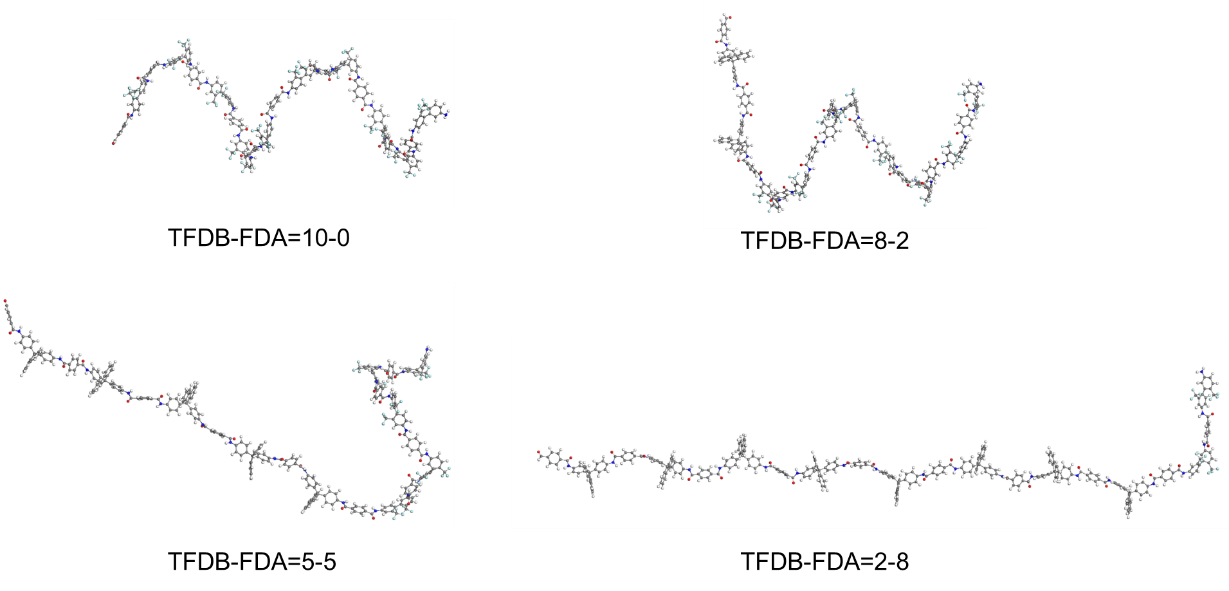


**Figure S2.** Effects of FDA contents upon the structural rigidity of the polymer chain simulated with molecular dynamics.


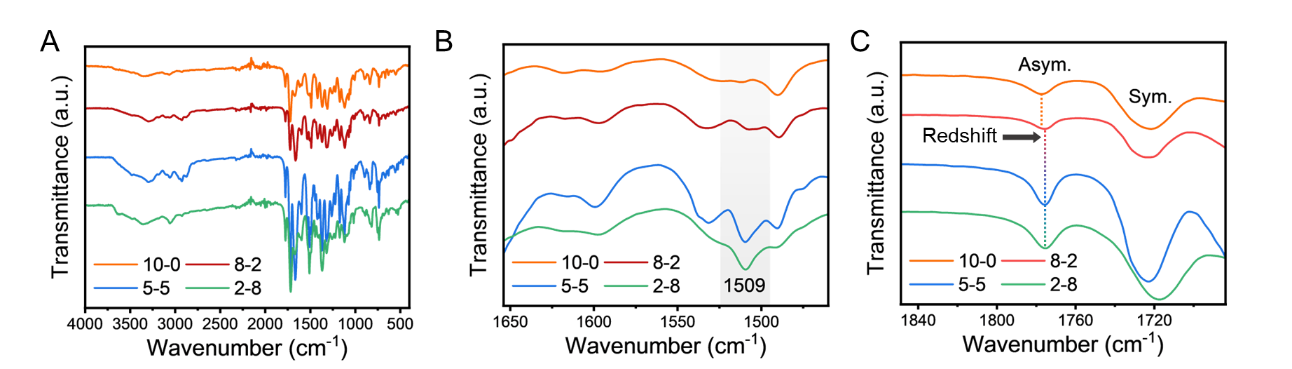


**Figure S3.** Fourier transformed infrared (FTIR) patterns of PAIs. a) Full spectra. b) Increased intensity of the characteristic peak of FDA as its content increases. c) FTIR peaks corresponding to the symmetric and asymmetric stretch of C=O in the imide ring.


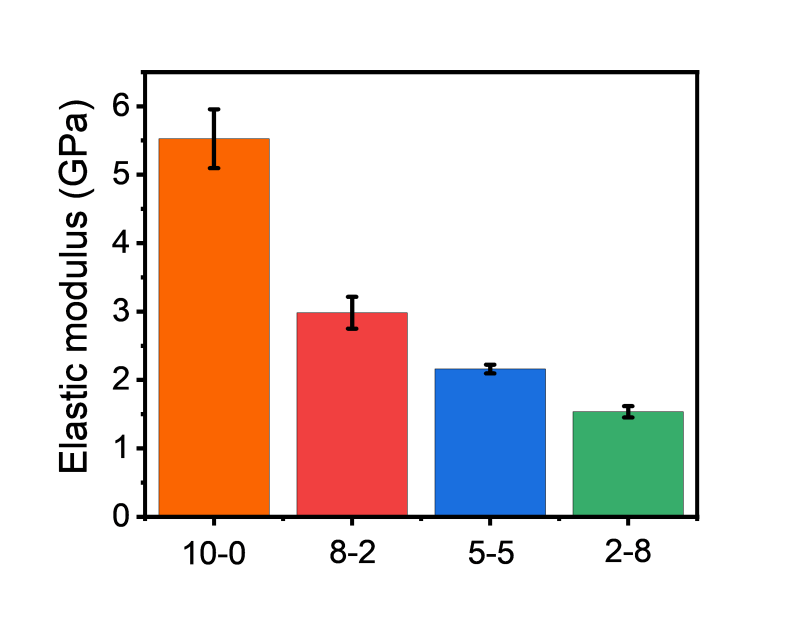


**Figure S4.** Dependence of tensile modulus upon the composition of PAI.


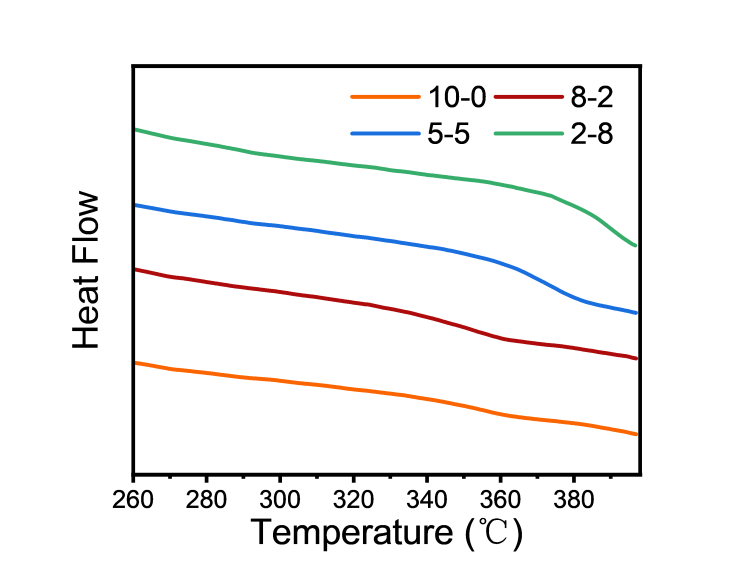


**Figure S5.** DSC curves of PAIs with different TFDB-FDA ratios.


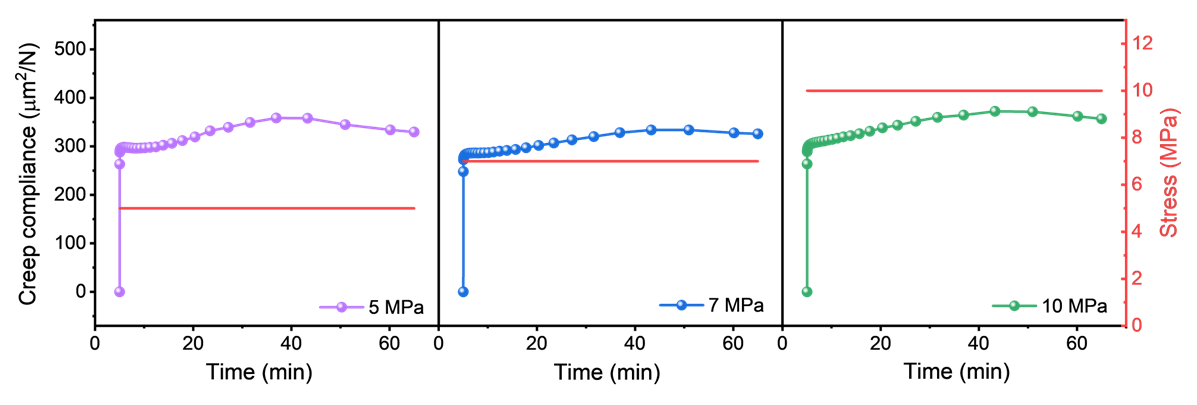


**Figure S6.** Creep curves of PAI 5-5 under different stresses at 30℃.


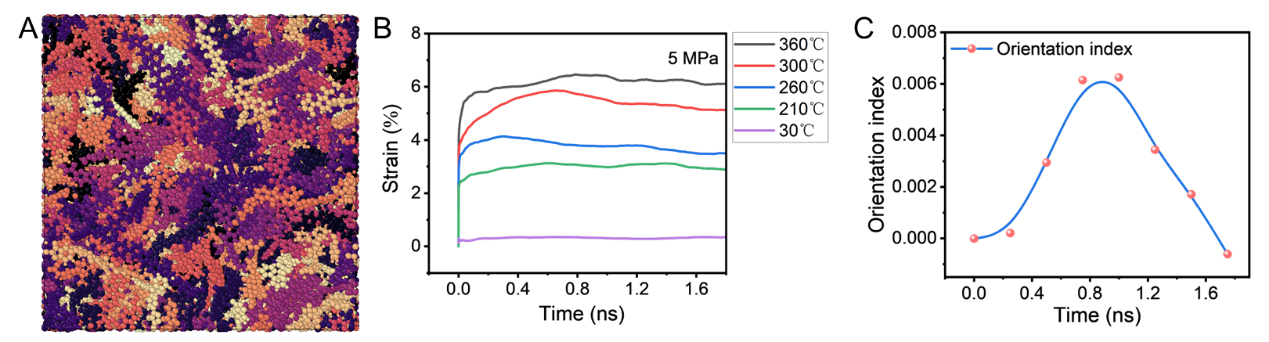


**Figure S7.** a) PAI 5-5 model constructed in molecular dynamics (MD) simulation. b) Creep curves of PAI 5-5 obtained in MD simulations. c) Orientation index of PAI 5-5 molecular chains corresponding to the creep curve of 300℃ in b). A higher orientation index indicates a higher orientation level of polymer chains.


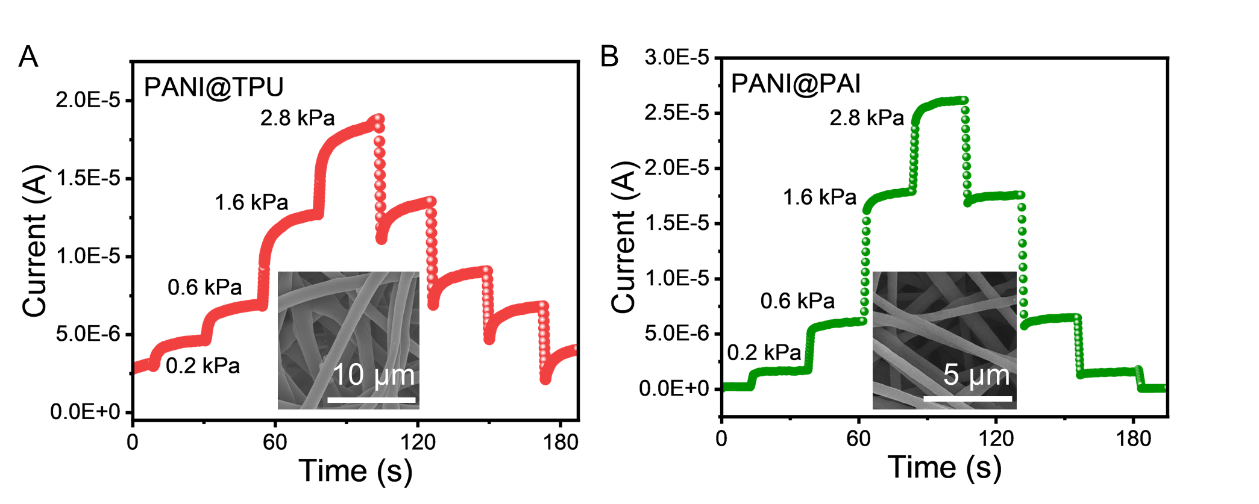


**Figure S8.** Comparison of the stability and recoverability of pressure sensors based on conventional a) elastomeric TPU and b) anti-creep PAI.


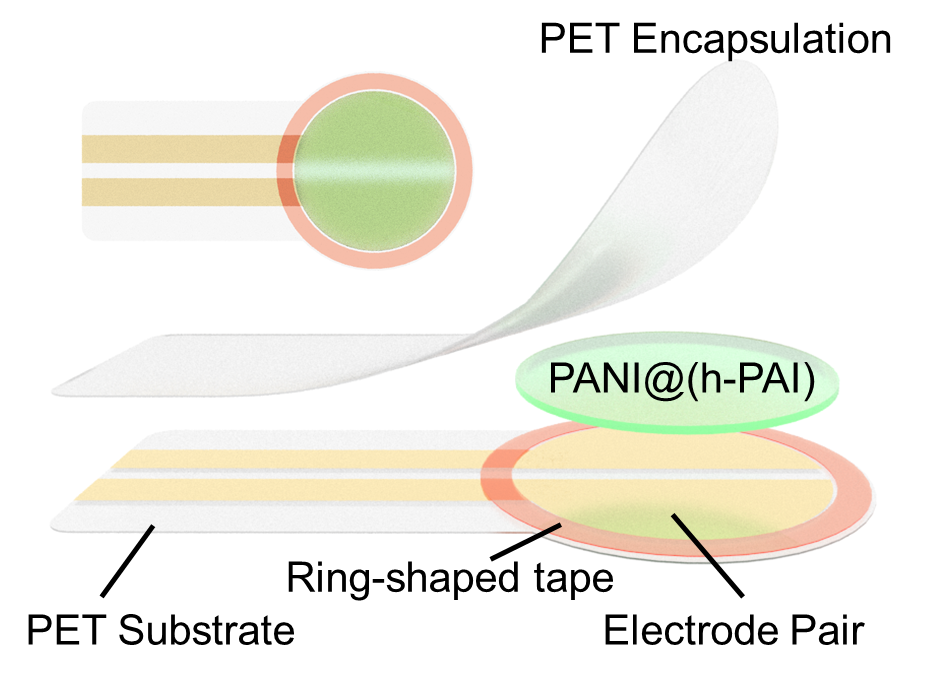


**Figure S9.** Construction of encapsulated pressure sensors based on PANI@(h-PAI).


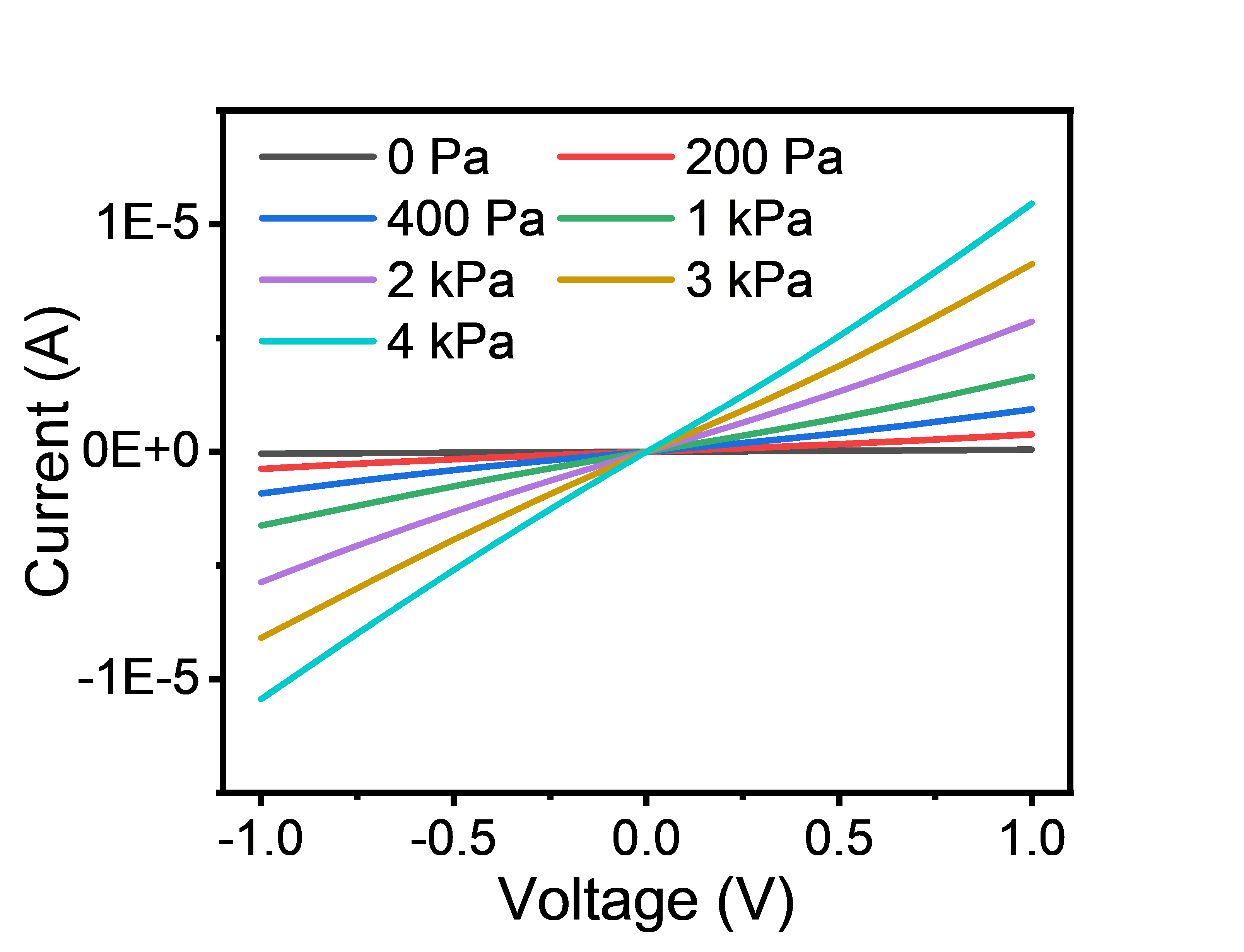


**Figure S10.** Voltage sweeping curves of PANI@(h-PAI).


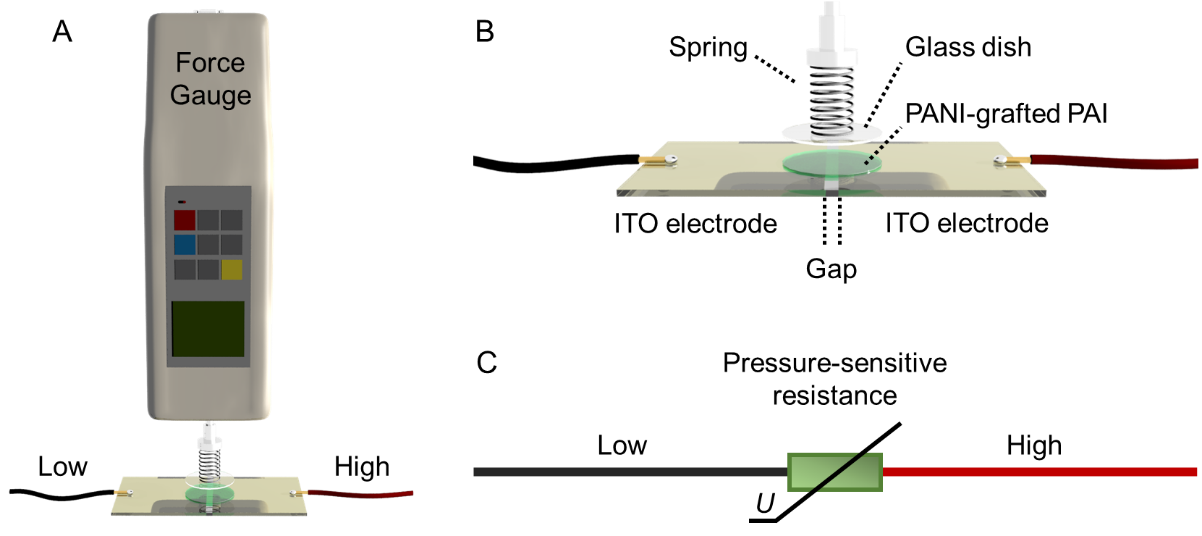


**Figure S11.** a) Experimental setup for the measurement of sensitivity. b-c) Illustration of the electrical circuit.


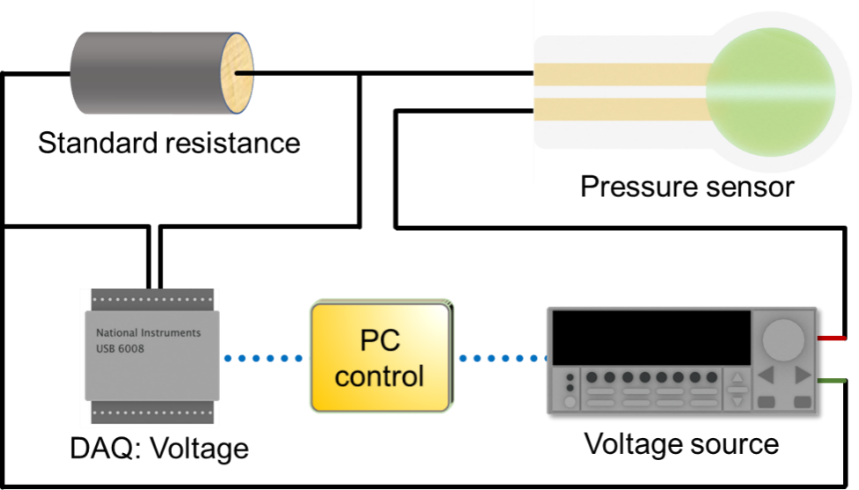


**Figure S12.** Experimental setup for the measurement of response time.


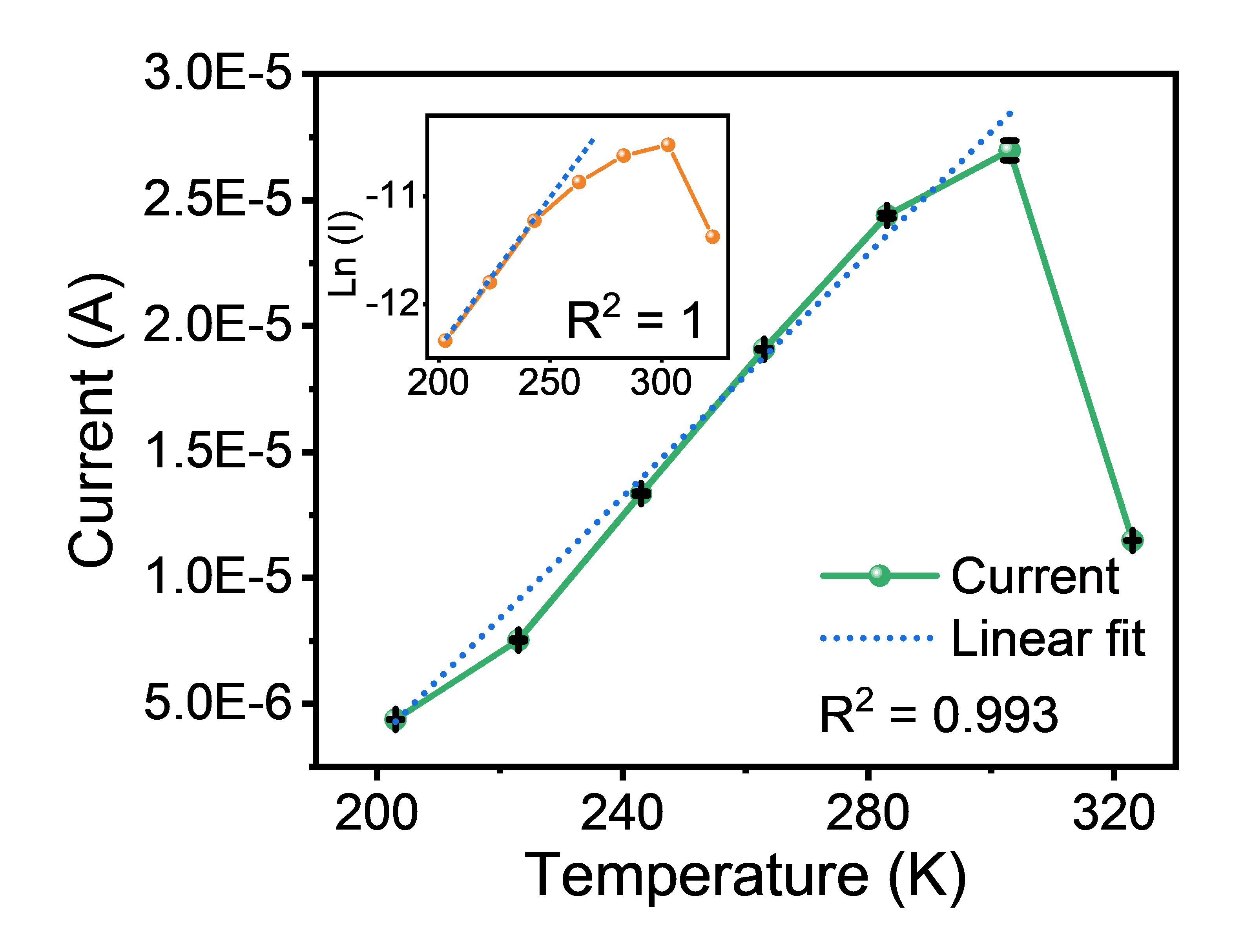


**Figure S13.** Temperature dependence of the current signal.


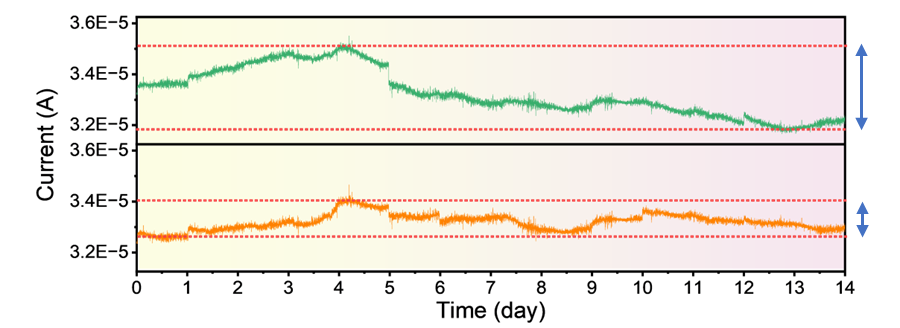


**Figure S14.** The effect of temperature compensation decreases temperature-induced signal deviation.


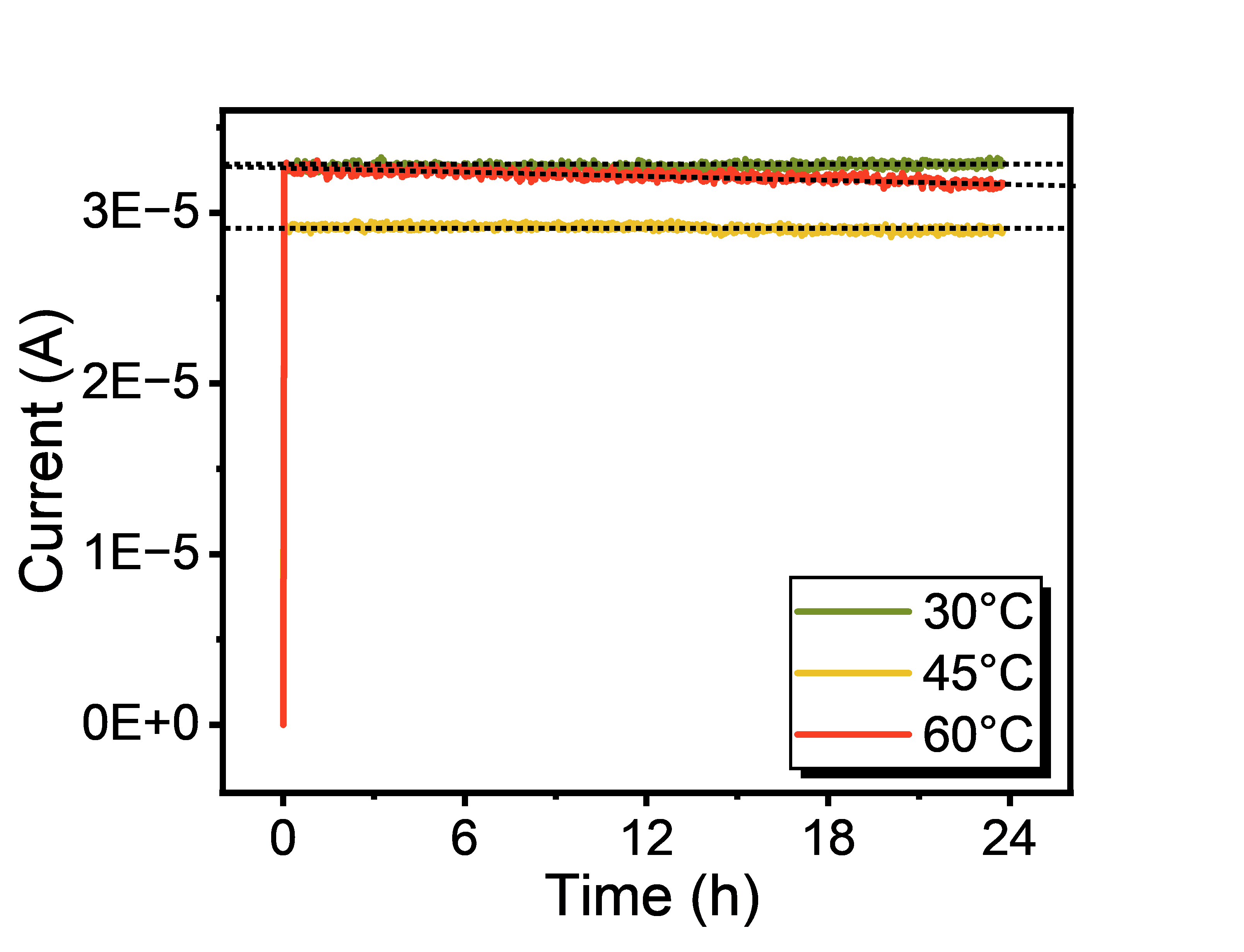


**Figure S15.** Static stability under different temperatures.


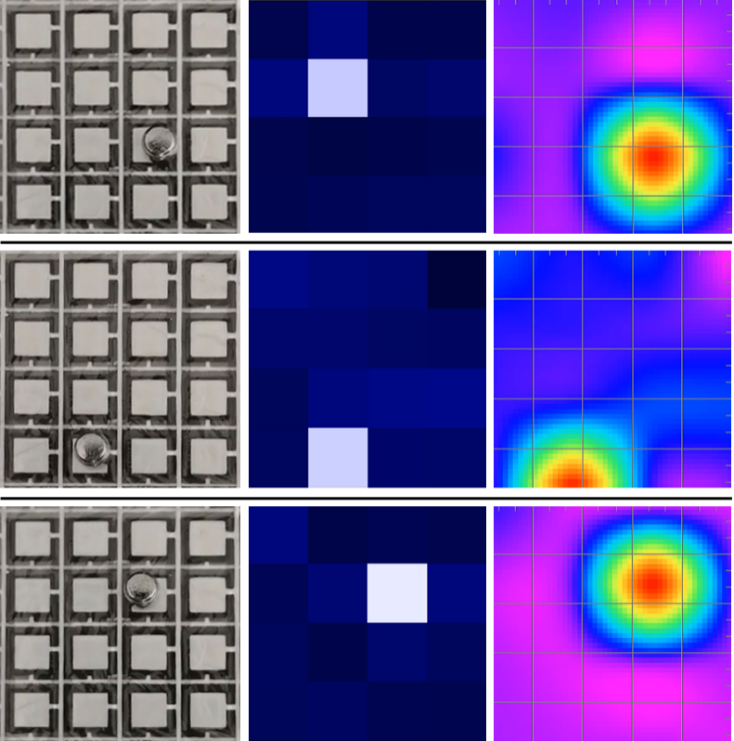


**Figure S16**. Capability of the pressure sensor matrix to detect the movement of a standard weight (1 g).


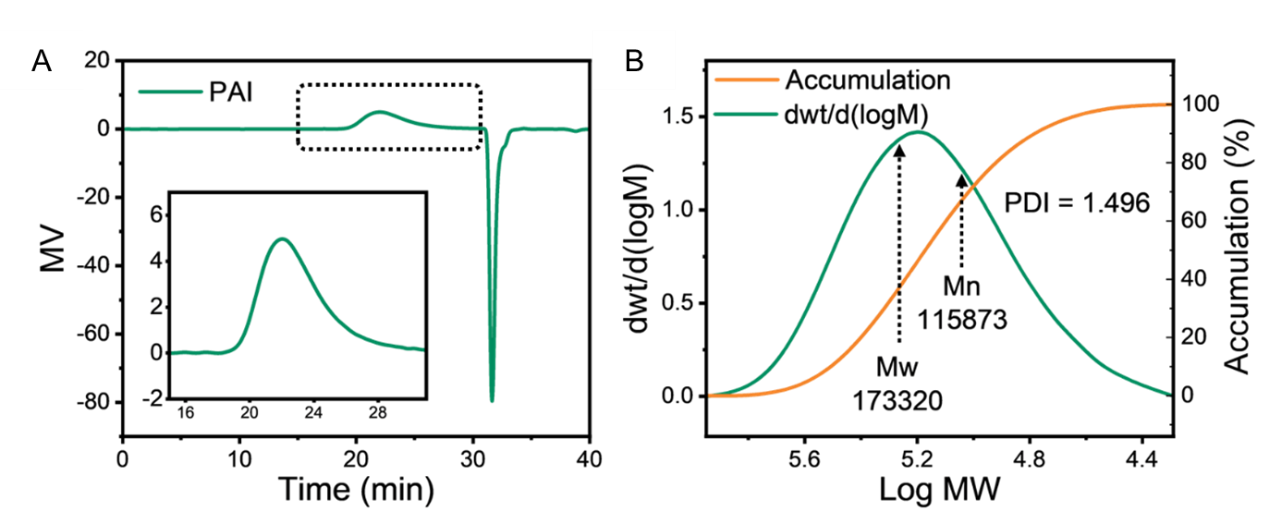


**Figure S17.** Molecular weight of as-synthesized PAI (5-5).


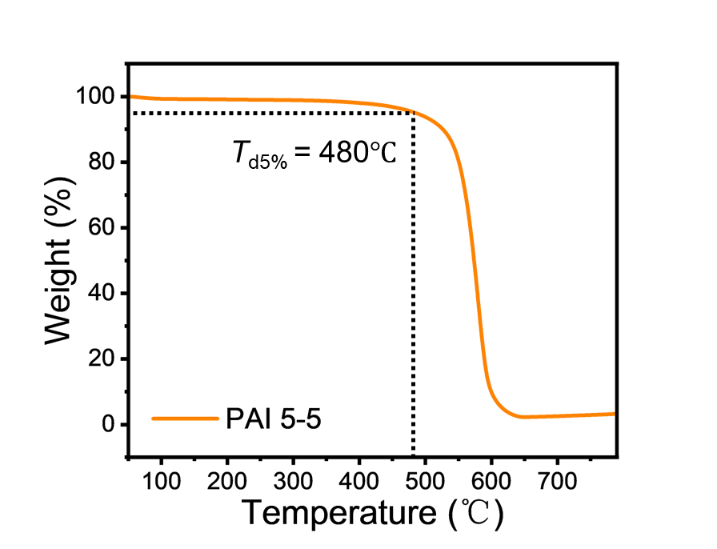


**Figure S18.** Thermogravimetric curve of PAI (5-5).


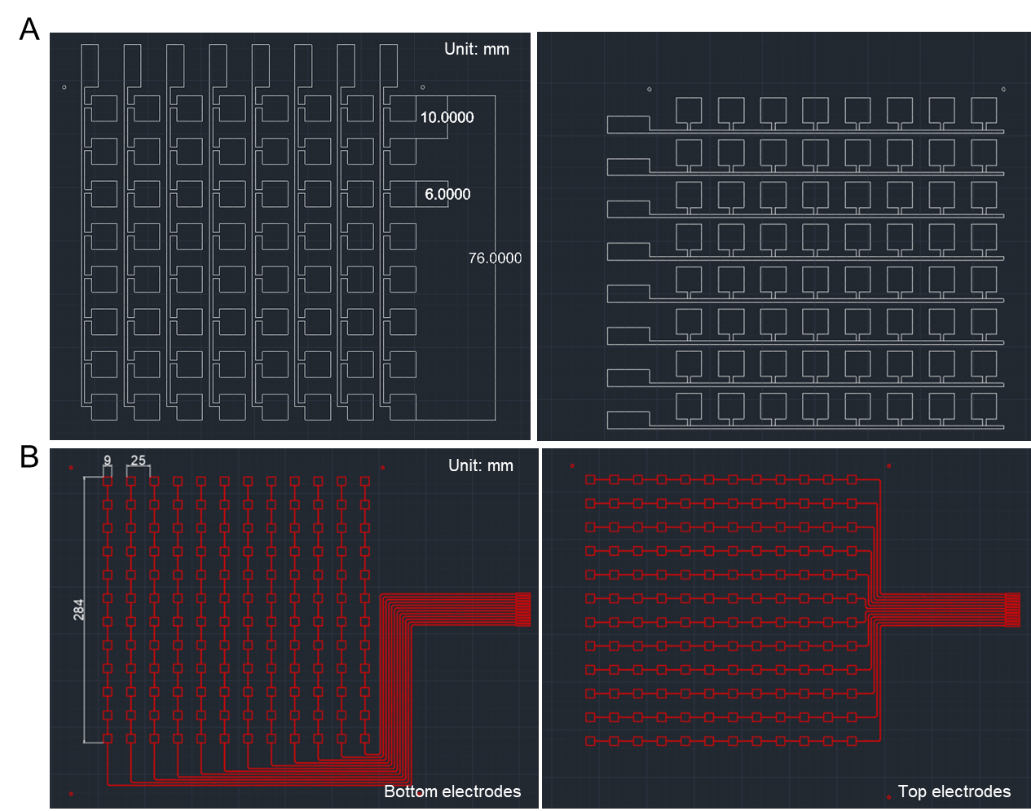


**Figure S19.** Dimensional scale of the electrode array. a) 8 × 8 for gesture recognition. b) 12 × 12 for seating posture recognition.

**Table S1.** Peak assignment of the FTIR spectrum of PAI.

| Position (cm^-1^) | Assigned group |
| --- | --- |
| 1775 | Asymmetric C=O of imide |
| 1722 | Symmetric C=O of imide |
| 3295 | N-H |
| 1666 | C=O of amide |
| 1369 | C-N |
| 1321 | C-F |
| 1304 | C-F |

**Table S2.** Peak assignment of the FTIR spectrum of PANI@PAI.

| Position (cm^-1^) | Assigned group |
| --- | --- |
| Around 3250 | N-H stretching mode |
| 1568 | C=N quinoid ring stretching |
| 1500 | C=C benzenoid ring stretching |
| 1307, 1250 | C-N stretching of benzenoid units |
| 1150, 1125 | C-N stretching of benzenoid units |
